# Kinetics Crystallization and Polymorphism of Cocoa Butter throughout the Spontaneous Fermentation Process

**DOI:** 10.3390/foods11121769

**Published:** 2022-06-15

**Authors:** Efraín M. Castro-Alayo, Llisela Torrejón-Valqui, Marleni Medina-Mendoza, Ilse S. Cayo-Colca, Fiorella P. Cárdenas-Toro

**Affiliations:** 1 Instituto de Investigación, Innovación y Desarrollo para el Sector Agrario y Agroindustrial de la Región Amazonas (IIDAA), Facultad de Ingeniería y Ciencias Agrarias, Universidad Nacional Toribio Rodríguez de Mendoza de Amazonas, Calle Higos Urco 342-350-356, Chachapoyas 01001, Amazonas, Peru; llisela.torrejon@untrm.edu.pe (L.T.-V.); marleni.medina.epg@untrm.edu.pe (M.M.-M.); 2 Programa de Doctorado en Ingeniería, Departamento de Ingeniería, Pontificia Universidad Católica del Perú, Av. Universitaria 1801, San Miguel 15088, Lima 32, Peru; 3 Sección de Ingeniería Industrial, Departamento de Ingeniería, Pontificia Universidad Católica del Perú, Av. Universitaria 1801, San Miguel 15088, Lima 32, Peru; fcardenas@pucp.pe; 4 Facultad de Ingeniería Zootecnista, Agronegocios y Biotecnología, Universidad Nacional Toribio Rodríguez de Mendoza de Amazonas, Calle Higos Urco 342-350-356, Chachapoyas 01001, Amazonas, Peru; icayo.fizab@untrm.edu.pe

**Keywords:** criollo cocoa, cocoa butter, polymorphism, crystallization, differential calorimetry scanning, Avrami model, kinetics

## Abstract

The spontaneous fermentation process of Criollo cocoa is studied for its importance in the development of chocolate aroma precursors. This research supports the importance of spontaneous fermentation, which was studied through the crystallization behavior and polymorphisms of cocoa butter (CB), the most abundant component of chocolate that is responsible for its quality physical properties. The k-means technique was used with the CB crystallization kinetics parameters to observe the division of the process during the first stage (day 0–3). The experimental crystallization time was 15.78 min and the second stage (day 4–7) was 17.88 min. The Avrami index (1.2–2.94) showed that the CB crystallizes in the form of a rod/needle/fiber or plate throughout the process. CB produced metastable crystals of polyforms $\beta_{1}^{\prime }$ and $\beta_{2}^{\prime }$. Three days of fermentation are proposed to generate Criollo cocoa beans with acceptable CB crystallization times.

## 1. Introduction

Cocoa butter (CB) is a natural fat extracted from cocoa beans [1]. CB has a crystalline structure that determines the final quality of chocolate [2,3]. CB has a crystal acylglycerols (TAGs) [4,5] that define its crystallization properties [6,7,8]. CB is mainly composed of 23.2–29.3% 1,3-di-stearoyl-2-oleoyl-glycerol (SOS), 34.2–38.6% 1-palmitoyl-2-oleoyl-3-stearoyl-glycerol (POS), and 13.5–17.1% 1,3-dipalmitoyl-2-oleoylglycerol (POP) [9,10]. These TAGs allow CB to crystallize in six polyforms, which are γ, α, $\beta_{2}^{\prime }$′, $\beta_{1}^{\prime }$, $\beta_{2}$, and $\beta_{1}$, and are identified according to their melting temperatures (17.3, 23.3, 25.5, 27.5, 33.8, and 36.3 °C; respectively) [9,11,12,13,14] and stability. Although the contribution of POP is less than those of POS and SOS, its presence may influence the formation of metastable polymorphic forms in CB [15]. The polymorphism is related to the organoleptic and physical characteristics of the chocolate [16]. That means that CB crystals define the sensory characteristics of chocolate, such as flavor release, mouthfeel, and melting properties [3]. Characterizing these crystalline forms is crucial to meeting market needs, optimizing processes, or developing new products [17]. Therefore, CB crystallization is an important phenomenon that must be considered to obtain high-quality chocolates [18]. Obtaining the $\beta_{2}$ polyform (form V) is very important for chocolate manufacturers [8,17,19,20,21] due to it giving the optimal characteristics of gloss, snap, texture, melting, sensation in the mouth, and bloom resistance [4,20,22].

In the chocolate manufacturing chain, the fermentation of cocoa beans is essential for the generation of aromatic compounds in chocolate [23,24,25] and other metabolites, such as amino acids, amines [26], and polyphenols [27], generated by biochemical reactions that are favored by temperature, pH, and time [28]. This process occurs spontaneously on farmers’ farms, unlike other fermentation processes [29]. The fermentation methods used and the action of indigenous microorganisms result in a very heterogeneous process [29]. Therefore, controlling the process variables is necessary to obtain cocoa of better quality and homogeneity [28]. A widely studied fermentation process is acidification by acetic acid (the corresponding pH is a variable), whose action produces biochemical modifications related to chocolate flavor [30]. Although they could also be related to the crystallization behavior of CB during fermentation, there is not enough evidence for it [5,31]. Moreover, no studies have analyzed the crystallization of CB in its natural state during spontaneous fermentation (when CB is under the influence of other chemical components of the cocoa bean). We believe that the closest test to reality would be to study the crystallization of CB inside the cocoa bean without being influenced by any extraction method.

The most used method to describe the behavior of cocoa butter is the Avrami model, which describes the kinetics of crystallization and crystal growth [1,32,33]. Avrami’s equation describes an initial period where crystallization occurs slowly and is followed by a rapid increase in the mass of the crystal formed. It is assumed that this process occurs under isothermal conditions, and crystal growth is by random nucleation and linear growth [34,35,36]. On the other hand, the analytical technique of X-ray diffraction (XRD) is the most used to study the polymorphism of fat crystals. However, access to XRD equipment is not easy in a research and development unit, so differential scanning calorimetry (DSC) [12] is an alternative. MacNaughtan et al. [37] demonstrated that the DSC technique could provide reproducible kinetic data on the tristearin-tripalmitin crystallization and polymorphism. They also provided the identity of the polymorphs after the fusion of the crystallized material. Similarly, Simoes et al. [38] used DSC to study the polymorphic transition of CB from form $\beta_{1}^{\prime }$ to $\beta_{2}$, in the presence of emulsifiers and sugar.

Optimizing the fermentation process to obtain good quality cocoa beans involves studying the metabolites generated [39]. However, we also consider it necessary to describe the fermentation process based on the quantitative crystallization kinetics and CB polymorphism. Therefore, the objective of this work was to describe the kinetics of crystallization and polymorphism of CB throughout the spontaneous fermentation process.

## 2. Materials and Methods

### 2.1. Materials and Chemicals

Fresh Criollo cocoa beans and pure CB were provided by Cooperativa de Servicios Múltiples Aprocam (Bagua–Amazonas–Peru). The 1,3-dipalmitoyl-2-oleoylglycerol (POP) ≥99% standard was purchased from Sigma Aldrich.

### 2.2. Monitoring Fermentation

Considering the work carried out by Deus et al. [26], the spontaneous fermentation of Criollo cocoa was carried out in the Aprocam cocoa processing center. The cocoa pods were harvested and opened with a stainless-steel knife. The beans, surrounded by their pulp, were placed in polypropylene bags, taken to the processing center, and placed in 50 × 50 × 50 cm wooden boxes (40 kg capacity). The boxes were covered with jute bags to prevent the bean mass from losing heat. The bean mass was passed from one box to another to allow aeration. During each day of fermentation, the temperature and pH parameters were recorded. Cocoa bean samples (50 g) were extracted daily from the total mass, and we recorded the start time (Day 0) and end time of fermentation (day 7). The samples were packed in sterile polypropylene bags and stored in liquid nitrogen until they were taken to the UNTRM laboratory for physicochemical analysis in duplicate.

### 2.3. Physico-Chemical Parameters

#### 2.3.1. Titratable Acidity and pH of the Cocoa Beans

The titratable acidity and pH were determined according to the AOAC 942.15 and 970.21 methods [40].

#### 2.3.2. Moisture Content

According to Elbl et al. [41], with modifications, the moisture content of cocoa beans was determined using a halogen moisture analyzer (Mettler Toledo, Excellence plus HX204, Greifensee, Switzerland) based on the gravimetric principle. One gram of sample was weighed in an analyzer crucible and heated to 105 °C until a constant weight was obtained. The difference in weight (initial and final) calculated by the analyzer gave the moisture content.

#### 2.3.3. Water Activity

According to [42], water activity was determined with a portable water activity analyzer (Rotronic AG, HygroPalm-HP23-AW-A, Bassersdorf, Switzerland), and the sample container was filled with cocoa beans up to 4/5 of its capacity. The $A_{w}$ probe was placed immediately above the container. After 5 min, the AwQuick mode’s $A_{w}$ of the sample was recorded.

### 2.4. Crystallization and Melting Profiles of POP and CB Inside Cocoa Bean

According to Sonwai et al. [20] and Foubert et al. [43], with some modifications, the crystallization and melting profiles of POP and CB inside the cocoa beans were determined. A differential scanning calorimeter (DSC) (TA Instruments, Discovery DSC 2500, New Castle, DE, USA) was used. A 10–15 mg sample of cocoa bean was placed in an aluminum pan and hermetically sealed with the press. An empty pan was used as a reference. The sample was heated from room temperature to 60 °C and held for 15 min to ensure homogeneity and remove any crystal memory. The sample was cooled with a ramp of 5 °C/min to −20 °C and held for 15 min, and then heated at 5 °C/min to 60 °C. The Trios software determined the $T_{onset}$, $T_{endset}$, crystallization temperature $T_{c}$, melting temperature $T_{m}$, and enthalpy.

### 2.5. Isothermal Crystallization of Cocoa Butter Inside the Cocoa Bean

According to Toro-Vásquez et al. [44], Martini [31], and Ray et al. [11], from the $T_{c}$ and $T_{m}$ identified in 2.4, the experimental $T_{c}$ was chosen, considering that the crystallization process occurs at low supercooling (0–4 °C below the $T_{m}$ of the sample), so the $T_{c}$ used were: 15, 16, 17, 18, and 19 °C. The samples were cocoa beans collected on each day of spontaneous fermentation. A sample of pure CB was established as a control. A 10–15 mg sample of cocoa bean was placed in an aluminum pan and hermetically sealed. For the isothermal crystallization analysis and to erase any crystal memory, the sample was heated to 60 °C for 20 min. The sample was cooled with a ramp of 3 °C min^−1^ until reaching the correspondent $T_{c}$. Then, it was kept for 90 min until the exotherm (peak) of crystallization was completed. Next, the sample was heated at 5 °C min^−1^ up to 60 °C to obtain the melting endotherm (peak) that served to identify the polymorphic forms of CB, which were found by comparing the $T_{m}$ of the peaks with the literature. According to MacNaughtan et al. [37], reheating the sample does not influence its melting and crystallization behavior.

### 2.6. Kinetics Crystallization

The data from the isothermal crystallization were fitted to the Avrami equation (Equation (1)) [31,37,45] to calculate the crystallization kinetic parameters. The package Crystallization fit of Origin Pro Software created by Lorenzo et al. [46] was used.

(1) $$1-V_{c}\left(t\right)=\exp\left(-kt^{n}\right)$$

where $V_{c}$ is the fraction of crystal formed at time t during crystallization, k is the crystallization rate constant, and n is the Avrami index related to the crystallization mechanism [33]. Equation (1) is expressed in logarithmic form as:

(2) $$\ln\left[-\ln\left(1-V_{c}\left(t\right)\right)\right]=\ln k+n\ln t$$

From the plot of $\ln\left[-\ln\left(1-V_{c}\left(t\right)\right)\right]$ vs. lnt, n was calculated as the slope of the graph. This is the so-called Avrami plot. k is a function of the crystallization temperature and considers the crystal’s nucleation and crystal growth rate [34]. n indicates the crystal growth mechanism [33]; that is, it indicates whether nucleation is instantaneous (when the nuclei appear all at once when the process begins) or sporadic (when the number of nuclei increases linearly with time) and whether nuclei grow as rods, disks, or spheres (Table 1). Fractional values of n indicate the simultaneous generation of two or more types of crystals or similar crystals from different types of nuclei [34].

In addition to k and n, $t_{1/2}$ describes the time taken to achieve half of the overall crystallization and is calculated using Equation (3):

(3) $$t_{1/2}={\left(\frac{0.69315}{k}\right)}^{1/n}$$

Another important parameter is the crystallization induction time $t_{0}$. It is defined as the time required for the exotherm to begin to form [31] and calculated from the isothermal thermogram obtained by DSC. The $t_{0}$ is the time that elapses from the start of the isothermal process to the start of crystallization. This is the point of the thermogram where the sample’s heat fluxes deviate significantly from the baseline [47].

### 2.7. Polymorphism

The method developed by Fernandes et al. [18] was used to study the CB polymorphism during spontaneous fermentation. The sample was heated at 5 °C min^−1^ for each $T_{c}$ up to 60 °C until the crystals were melted (formed at the corresponding $T_{c}$). The polymorphic forms of CB were identified based on the $T_{m}$.

### 2.8. Statistical Analysis

Spontaneous fermentation samples were related to the BC crystallization behavior inside the cocoa bean, and unsupervised pattern recognition theory was used to form clusters using the k-means technique [48]. The isothermal crystallization kinetic parameters obtained by fitting the Avrami equation and the polymorphism results were analyzed by k-means cluster analysis using the RMarkdown software (Rstudio, version 2021.09.0+351, Boston, MA, USA) to find CB crystallization patterns within cocoa beans throughout the fermentation. As cluster analysis is an exploratory method, no replicates of the measurements [48] were used to run the k-means.

## 3. Results

### 3.1. Monitoring Fermentation

**Figure 1:**
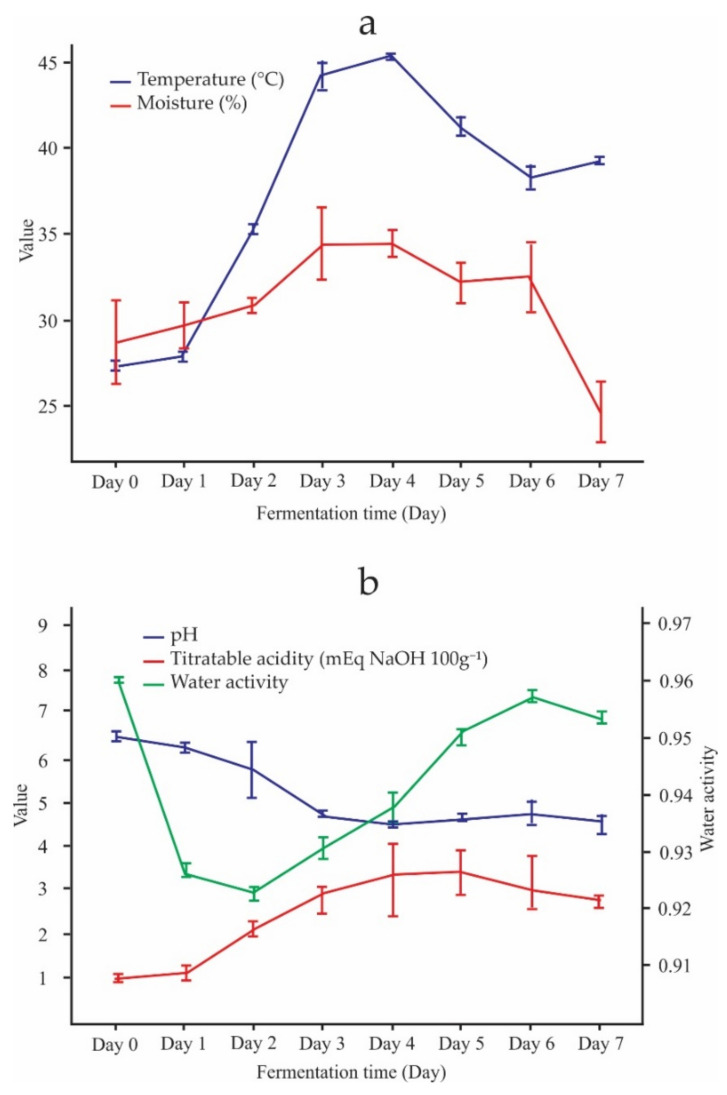
Evolution of spontaneous fermentation parameters of Criollo cocoa beans. (**a**) Evolution of temperature and moisture, (**b**) Evolution of pH, titratable acidity and water activity.

According to Figure 1, the parameters of the spontaneous fermentation of the Criollo cocoa beans showed variations concerning the days of fermentation. Figure 1a shows that the temperature increased as the fermentation developed until day 4 (45.35 °C) and then decreased towards the end of the process (39.2 °C). Similar behavior can be verified in cocoa bean moisture. Figure 1b shows that the pH and titratable acidity showed the opposite behavior. While the pH decreased with the days of fermentation (from 6.83 to 4.55), the acidity increased (from 1.15 to 2.8 mEq NaOH 100 g^−1^). Regarding the $A_{w}$, this parameter showed slight growth until the end of fermentation up to 0.95.

### 3.2. Crystallization and Melting Profiles

Table 2 shows CB’s crystallization and melting profiles compared to one of its main triglycerides, 1,3-dipalmitoyl-2-oleoylglycerol (POP). The CB started to crystallize at 15.45 °C, and $T_{c}$ was 12.93 °C. The POP began to crystallize at 12.07 °C, and $T_{c}$ was 10.40 °C. Crystallization enthalpies were 48.42 J/g for CB and 54.91 J/g for POP. The CB fusion range was 15.05 °C ($T_{onset}$) to 26.04 °C ($T_{endset}$), and the peak was at 19.65 °C ($T_{m}$); this range was lower for POP (12.75–19.34 °C), and its fusion peak was 15.75 °C. $T_{c}$ and $T_{m}$ of CB were used to establish the experimental $T_{c}$ for the kinetic crystallization, between 0 and 4 °C below $T_{m}$, thereby ensuring supercooling during isothermal kinetic crystallization. It can be seen that the melting enthalpy of CB (69.01 J/g) is lower than the melting enthalpy of POP (76.49 J/g).

### 3.3. Kinetics of Crystallization

**Figure 2:**
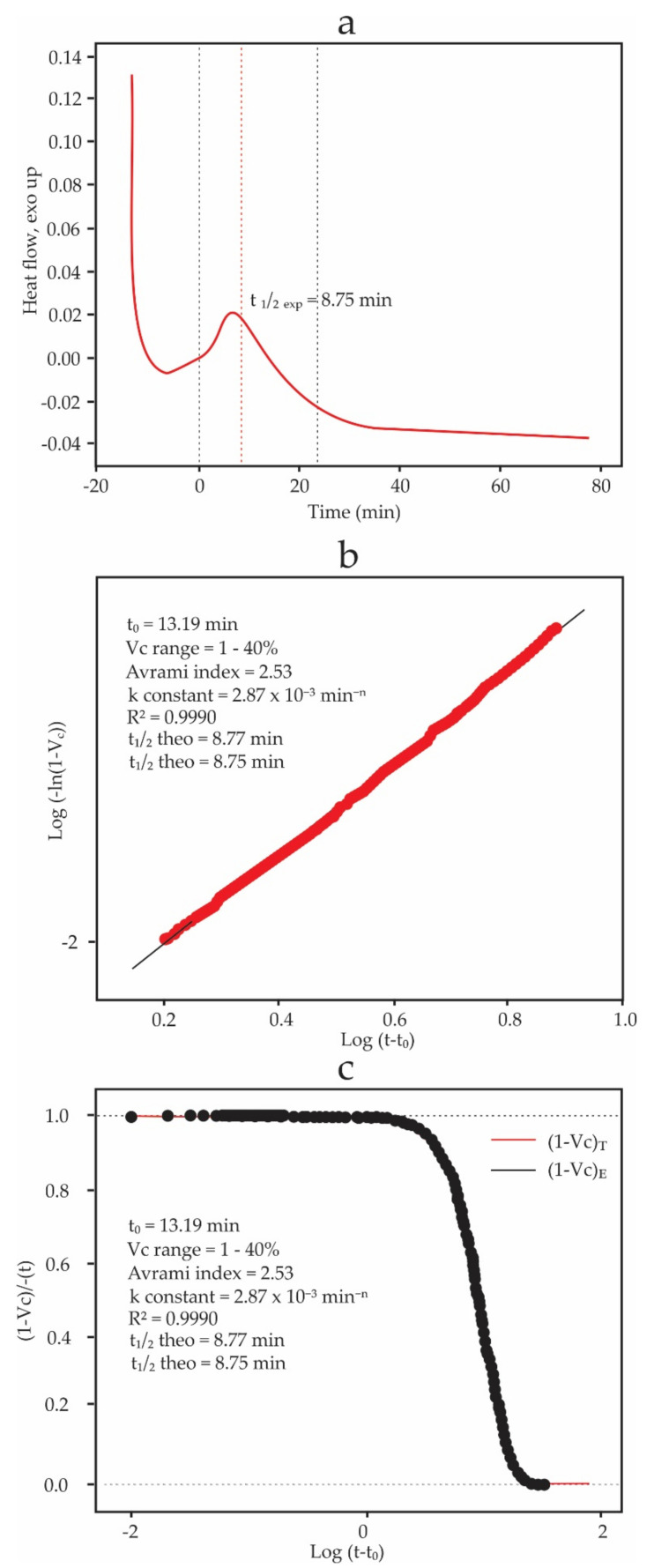
Results of fitting the CB isothermal crystallization data at 15 °C using the package Crystallization fit from Origin Pro. (**a**) Exothermal crystallization peak, (**b**) Avrami fitted range, (**c**) relative amorphous fraction.

Figure 2 shows the graphs resulting from fitting the CB isothermal crystallization experimental data at $T_{c}$ = 15 °C to the Avrami equation using the Crystallization fit package of Origin Pro software (Version: 9.8, Origin Lab, Northampton, MA, USA) 1 to 40% crystal conversion range was used, and the data fit of $R^{2}$ of 0.999 showed a good fit. The values of n and k were 2.53 and 2.87 × 10^−3^ min^−n^, respectively. The experimental half time, $t_{1/2}$, was 8.75 min. Figure 1a corresponds to the experimental data obtained by DSC. Figure 1b corresponds to the Avrami plot. Figure 2c shows the evolution of the untransformed fraction, $1-V_{c}$, as a function of time. POP and CB data crystallization at 15 °C can be found in the Supplementary Materials (Tables S1 and S2, respectively).

The results in Table 3 describe the kinetics of the crystallization of CB and POP. The CB n value increases as $T_{c}$ increases from 2.53 to 3.73. The opposite occurs with the value of k (from 2.87 × 10^−3^ to 8.54 × 10^−5^ min^−n^). The $t_{1/2}$ increase means that the CB will need more time to crystallize at a higher crystallization temperature. The same crystallization behavior has the POP. All data have a good fit, determined by the high values of $R^{2}$.

Table 4 shows the parameters of the Avrami equation at different $T_{c}$ for the study of the kinetics of crystallization of CB inside the cocoa bean during spontaneous fermentation. All the data have a good fit due to the high value of $R^{2}$. For each $T_{c}$, it is shown that a lower k generates higher theoretical and experimental $t_{1/2}$. Therefore, slower crystallization happens when crystallization occurred at 17, 18, and 19 °C and from day 4 of spontaneous fermentation. The values of n range from 1.2 to 2.94, indicating that the crystals grow in rod/needle/fiber or plate-like types.

**Figure 3:**
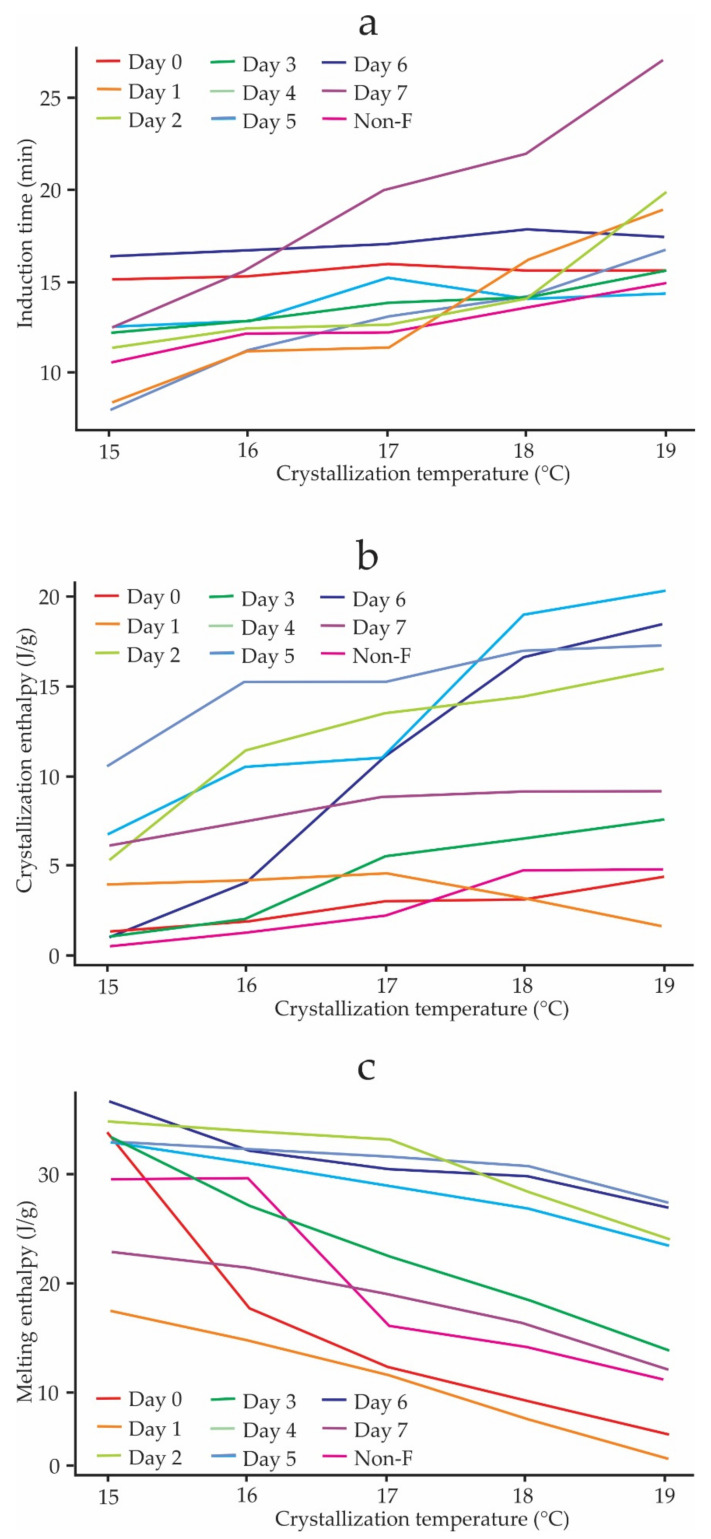
Induction times (**a**), crystallization enthalpy (**b**), and melting enthalpy (**c**) of the CB inside cocoa beans as a function of $T_{c}$ during spontaneous fermentation.

Figure 3a shows the induction times, $t_{0}$, for CB inside cocoa beans during spontaneous fermentation. The relationship of the $t_{0}$ is direct; that is, as the $T_{c}$ increases, the $t_{0}$ also increases. The highest $t_{0}$ corresponds to day 7 of fermentation, which means that the crystallization process started later on day seven than in the first days. Regarding the crystallization enthalpy, according to Figure 3b, this parameter shows a trend of increasing with $T_{c}$, generating the highest values at higher $T_{c}$. The opposite can be seen for the melting enthalpy (Figure 3c), which shows a decreasing trend with increasing $T_{c}$, presenting the lowest values on day 1 of fermentation.

**Figure 4:**
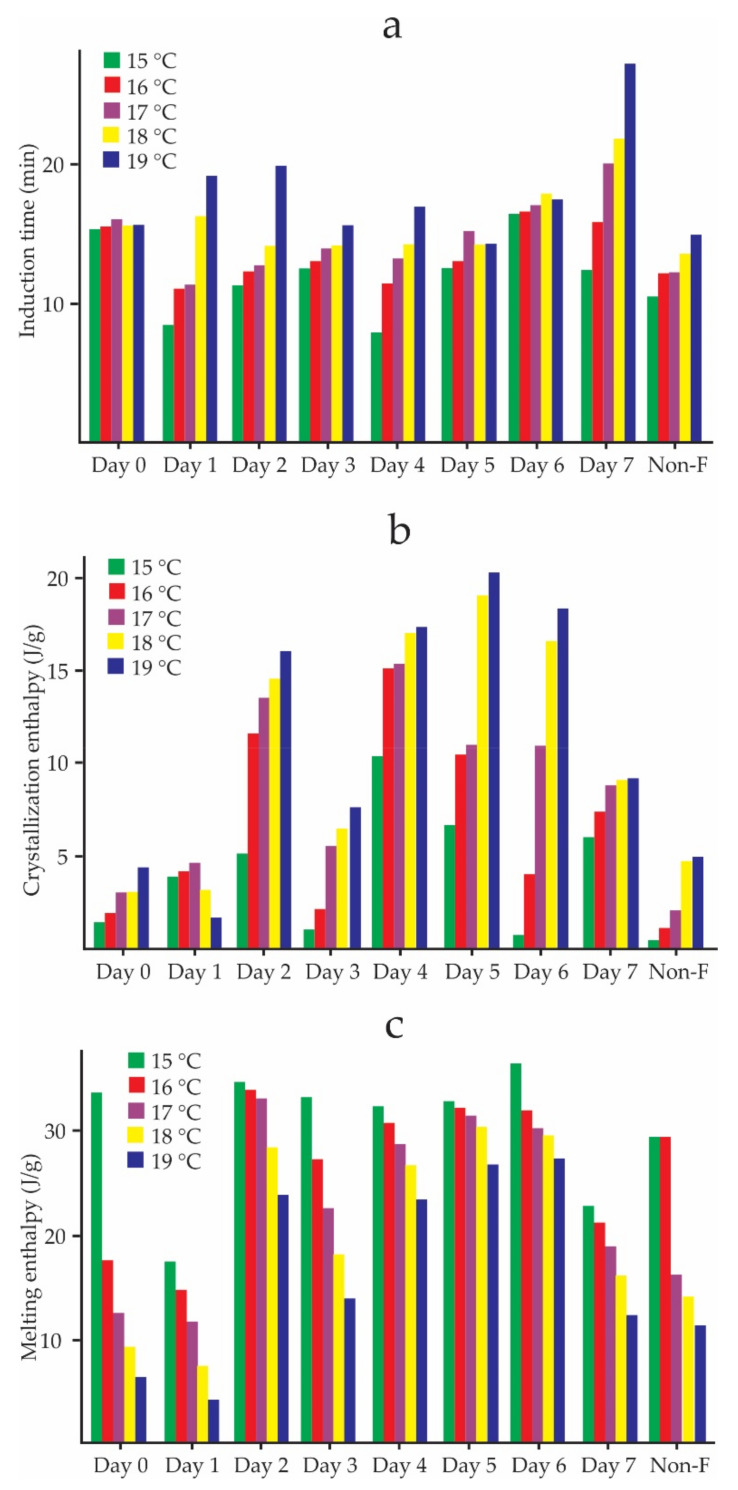
Induction times (**a**), crystallization enthalpy (**b**), and melting enthalpy (**c**) of the CB inside cocoa beans as a function of spontaneous fermentation days.

Figure 4a shows the evolution of the induction time, crystallization, and melting enthalpies from the point of view of the spontaneous fermentation process. The crystallization induction times of non-fermented cocoa beans were lower than those of fermented ones in all $T_{c}$ and increased each day of fermentation. The crystallization enthalpy reached its highest value on day 5 at 19 °C and had an increasing trend at each $T_{c}$. Non-fermented beans had the lowest values. Contrary to the enthalpy of crystallization, the melting enthalpy decreased with $T_{c}$, and had its highest values on day 6. The crystallization enthalpies were lower than the melting enthalpies during all days of fermentation.

**Figure 5:**
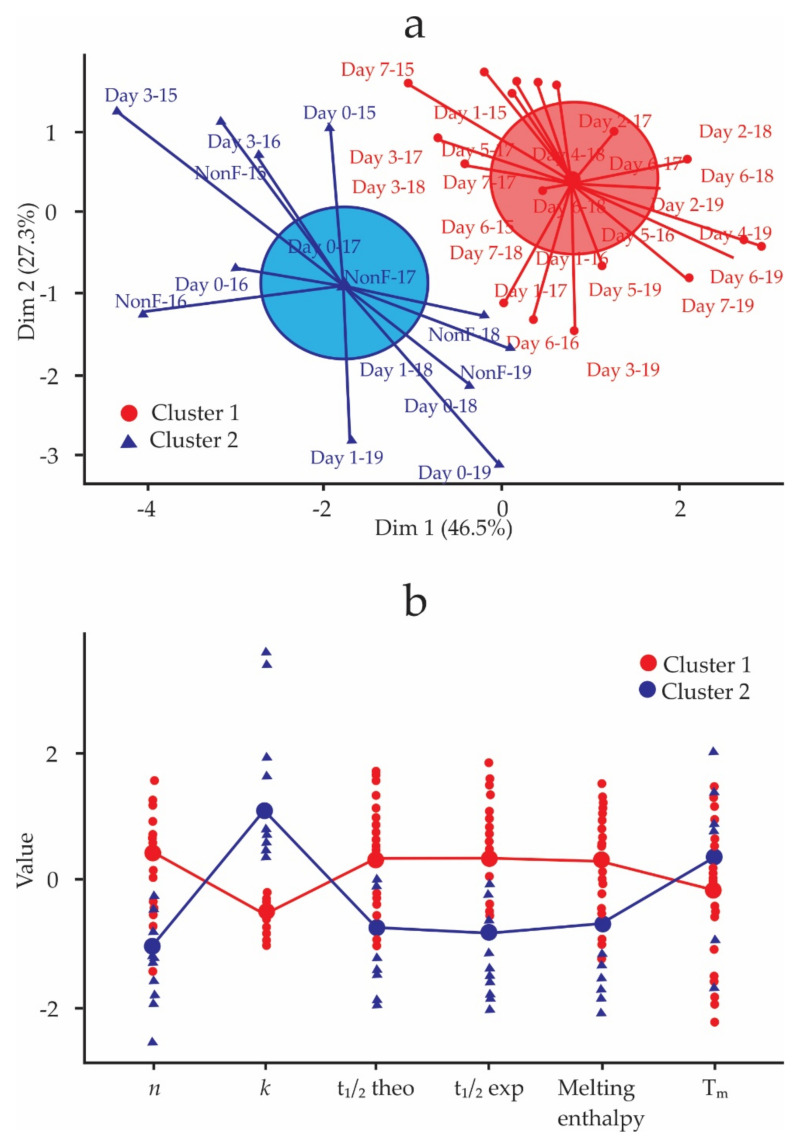
Clusters of kinetics parameters and polymorphic behavior of CB inside Criollo cocoa beans during spontaneous fermentation. (**a**) Division of the fermentation process into two stages (cluster), (**b**) Mean values of the crystallization kinetic parameters and polymorphism in each cluster.

Figure 5 shows the results of the k-means analysis: the ratio between the between-cluster sum of squares and the total sum of squares is 31.1%. In Figure 5a, the samples with a fermentation day higher than 4 make up cluster 1, and the samples with less than four days of fermentation and the non-fermented samples make up cluster 2. In Figure 5b, each cluster shows the means of the kinetics of crystallization and polymorphic behavior parameters. Cluster 1 is formed by the samples with the highest n values and the lowest k values related to the highest values of $t_{1/2}$. These samples also have higher melting enthalpies and lower $T_{m}$ values. The opposite occurs with cluster 2. These results mean that cluster 1 groups samples that crystallize more slowly with less stable polyforms and cluster 2 groups samples that crystallize faster with more stable polyforms. According to n, in cluster 1 are the samples whose crystals grew spherically and formed sporadic or instantaneous nuclei. Cluster 2 groups samples that crystallized as needles or fibers instantly or sporadically.

Table 5 shows the polymorphic behavior of CB and POP crystals at different $T_{c}$. The $T_{m}$ of all formed CB crystals increased directly with $T_{c}$, and the melting enthalpy decreased from 89.53 to 69.73 J/g. The melting range also increased. The $T_{c}$ used in the experiment produced a single polyform $\beta_{1}^{\prime }$ in the CB. In the case of the POP, the $T_{m}$, enthalpy and the melting interval had the same trend as the CB but with higher values, and the polyforms generated were of type α and $\beta_{2}^{\prime }$′.

Table 6 shows the polymorphism of CB inside cocoa beans throughout fermentation. The CB polymorphic forms were identified according to the sample $T_{m}$ after crystallization at a specific $T_{c}$. In all cases, the polymorphic forms identified were $\beta_{1}^{\prime }$ and $\beta_{2}^{\prime }$, which are thermally less stable. The melting ranges were between 19.06 and 30.35 °C. The melting enthalpies were higher when the crystallization was carried out at 15 °C than when it was carried out at 19 °C.

## 4. Discussion

### 4.1. Monitoring Fermentation

There was a slight decrease in temperature throughout the spontaneous fermentation of Criollo cocoa beans, from 45.35 °C on day 4 to 39.20 °C at the end of fermentation (Figure 1a). It could have been caused by the existing climatic conditions in the cocoa processing center of Aprocam. This behavior of the fermentation process is normal, if we compare it with the results obtained by Deus et al. [26] and Chagas Junior et al. [49]. Chagas Junior et al. [49] observed similar behavior in cocoa fermentation carried out in 50 kg boxes, as the temperature reached 37 °C after 7 days of spontaneous fermentation. This behavior coincides with the results reported by Visintin et al. [50]. Moisture also had the same behavior as temperature, reaching values of 24.6% until the end of fermentation (Figure 1a). In the spontaneous fermentation carried out by Deus et al. [26], decreasing of pH and increasing of the titratable acidity were observed, demonstrating that these behaviors are characteristic of a well-executed fermentation process [26,50,51,52]. Similar behavior was obtained during the spontaneous fermentation studied in this work. The $A_{w}$ increased slightly throughout the fermentation (Figure 1b).

### 4.2. Crystallization and Melting Profiles of CB and POP

The CB crystallization profile showed that its crystallization begins at a $T_{onset}$ of 15.45 °C and the peak is at $T_{c}$ of 12.93 °C (Table 2); these results that agree with those reported by Bayés-García et al. [53] ($T_{onset}$ = 16.6 °C and $T_{c}$ = 13.5 °C) and Aumpai [54] ($T_{onset}$ = 15.6 °C and $T_{c}$ = 10.6 °C). Since CB is a mixture of TAGs, we must talk about melting range instead of melting temperature [55]. Bayés-García et al. [56] reported the melting behavior at 2 °C/min of bulk CB and unfermented cocoa beans, finding forms α and $\beta_{2}^{\prime }$ ($T_{onset}$ and $T_{endset}$ of ∼17.5 and 28.6 °C, respectively), values close to those reported in Table 2. The difference in peak temperature is attributed to a different heating rate [57]. The POP crystallization results are according to Smith et al. [58]. The $T_{c}$ and $T_{m}$ of the POP are lower than those of the CB; however, their corresponding enthalpies are higher. The $T_{m}$ of CB and POP indicate the presence of unstable polyforms of type γ. Based on what was established by Sasaki et al. [15] and Ghazani [8], this polymorphic behavior of CB would be influenced mainly by POP.

### 4.3. Kinetics Crystallization

Lorenzo et al. [46] suggested that the isothermal crystallization data with an $R^{2}$ of 0.999 should be considered well adjusted, considering a conversion range according to the analyzed material. Fernandes et al. [18] used a conversion range of 1 to 40% for the crystallization kinetics of chocolate. This same range was used in the present work, resulting in a good fit of the CB crystallization data that exceeded the value of 0.999, and the graphs that characterize the Avrami equation were obtained (Figure 2). Figure 2a shows CB crystallization’s exothermic peak (exotherm); half of the crystallization was reached in 8.75 min. From this time, the secondary crystallization commenced. The initial descent in Figure 2a corresponds to the thermal stabilization time of the DSC, and $t_{0}$ was calculated from the stabilization time until t = 0. The induction time is understood as the time necessary to initiate crystallization [46]. Figure 2b shows a linear fit of the experimental data, from which the slope and the intercept have been determined to obtain the parameters of the Avrami equation (Equation (1)). Considering a conversion range for $V_{c}$ from 1 to 40% [18], an $R^{2}$ of 0.999 has been obtained, which shows a good data fit. Figure 2c compares the experimental and predicted values of the relative untransformed fraction as functions of time. The data show an excellent fit throughout the crystallization process, even beyond the primary crystallization.

The Avrami index n describes the forms in which crystal growth occurs (Table 1) [45,59]. According to Table 1, n can take values from 1 to 4. The CB’s values of n increased from 2.53 at 15 °C to 3.73 at 19 °C. The crystal nuclei can grow from plate-like to spherical as $T_{c}$ increases. POP’s n values were reduced from 3.07 to 2.61 as the Tc increased, indicating that its crystal nuclei’s growth behaves oppositely to that of CB.

On the other hand, decimal values of n indicate that both forms of crystal growth can co-occur, either sporadically or instantaneously. In the study carried out by Badu et al. [1], small values of n were obtained for Allanblackia seed oil and shea nut oil, which indicates their rapid nucleation and crystallization mechanism. The crystallization rate constant, k, of CB was higher than that of POP and increased with $T_{c}$; therefore, its crystallization is faster. This is demonstrated by the low $t_{1/2}$ values of the CB concerning the $t_{1/2}$ values of the POP.

Non-fermented cocoa beans show n values ranging from 2.13 at 15 °C to 2.17 at 19 °C (Table 4), and within them, CB crystals grow as rod/needle/fiber and plate. As fermentation began, the n values of the fermented cocoa beans changed every day from 1.20 to 2.94. However, they did not reach values similar to those of the CB (n = 3.73, spherical nuclei), indicating that the crystals grew the same as those of non-fermented beans. Crystallization kinetics experiments also showed that the parameters k, $t_{1/2}\;exp$, and $t_{1/2}\;theo$ changed throughout the fermentation process for all temperatures tested; i.e., the rate constant k was reduced, causing $t_{1/2}$ to increase, hence a slower CB crystallization process as the fermentation progressed. This behavior is due to the chemical composition of the cocoa bean, which changes throughout fermentation by the generation of other components that influence the crystallization behavior of CB [11,60,61].

The induction time, $t_{0}$, is a function of the $T_{c}$ [11,47,55]. Figure 3a shows an increasing trend of $t_{0}$ with $T_{c}$, being higher on day 7 at 19 °C (Figure 4a), which indicates that the crystallization of CB begins later in the last days of fermentation. This same trend was reported by Martini et al. [62] in the study carried out on anhydrous milk fat crystallization. Additionally, in this work, a decreasing trend of crystallization enthalpy concerning $T_{c}$ was reported [62], contrary to what can be observed in Figure 3a, which shows an increasing trend, and whose highest values occur in the intermediate days of fermentation (Figure 4b). The unfermented cocoa beans had the lowest crystallization enthalpy values during all the days of fermentation. Figure 3c shows a decrease in the melting enthalpies of crystallized CB within the cocoa beans at different temperatures, and the lowest values occurred on day 1 of fermentation (Figure 4c). This happened because of the driving force (supercooling) decreases with increasing crystallization temperatures [63].

Deus et al. [26] used cluster analysis to divide the spontaneous fermentation of cocoa into two stages: the first grouped the first days of fermentation, characterized by high levels of pH and free amino acids; the second stage grouped the last days of fermentation, characterized by high temperatures, high total titratable acidity, and high levels of free bioactive amines. The unsupervised k-means classification method [64] applied to the CB crystallization kinetics parameters divided the fermentation into two stages (cluster) (Figure 5a). The first corresponds to day 0 to day 3 and the second from day 4 to day 7. These findings reinforce the statements of Castro-Alayo et al. [51] and Cevallos-Cevallos et al. [65], who mention that Criollo cocoa requires fermentation for 2 or 3 days. In this work, we established that a fermentation process that exceeds 4 days will produce cocoa beans in which the CB will crystallize more slowly later than in the first 3 days. In addition to that, such a fermentation will produce less stable polyforms of type $\beta_{1}^{\prime }$ and $\beta_{2}^{\prime }$.

### 4.4. Polymorphism

It is crucial to study the polymorphic transitions of the CB because they influence the properties of chocolate [61]. The stability and melting temperatures ($T_{m}$) of the CB polyforms increase when they are transformed from γ to $\beta_{1}$. This irreversible transformation of phases depends on time and temperature, from the least stable to the most stable [18]. As mobility within the system increases due to a higher $T_{c}$, more stable polyforms are created without starting from the initial polyforms [55]. Crystals of form $\beta_{1}^{\prime }$ and $\beta_{2}^{\prime }$ are considered metastable [66]. In the case of CB, for all the $T_{c}$, only $\beta_{1}^{\prime }$ polyforms were formed (Table 5). The increase in $T_{c}$ (15–19 °C) was insufficient to generate polymorphism from the less stable forms to the more stable ones. To achieve this, more considerable increases in $T_{c}$ are necessary, according to what was reported by Fernandes et al. [18]. Similar behavior was noted in the POP for $T_{c}$ values from 17 to 19 °C, where only the $\beta_{1}^{\prime }$ polyform was generated. However, at 15 °C, polyform α was generated due to its low melting point. The melting enthalpies of the crystallized polyforms of CB and POP decreased with $T_{c}$. For any $T_{c}$, the melting enthalpy of the POP is always greater than that corresponding to CB.

In the same way as the previous results, there was no evidence of any change in the CB polymorphism towards more stable polyforms as fermentation went on (Table 6). This means that, during fermentation and at any $T_{c}$, only metastable CB polyforms ($\beta_{1}^{\prime }$ and $\beta_{2}^{\prime }$) are formed within the cocoa beans. The melting enthalpies of these polymorphic crystals are lower than those corresponding to the enthalpies of pure CB throughout the entire fermentation process. The results of Table 4 and Table 6 agree with what was established by Garbolino et al. [67], who state that the different results of the crystallization kinetics do not always produce changes in the polymorphism.

## 5. Conclusions

The present work allowed us to confirm the importance of the spontaneous fermentation process of Criollo cocoa analyzed from the point of view of the crystallization of cocoa butter. The parameters describing the kinetic crystallization of cocoa butter inside the Criollo cocoa bean show that only metastable crystals corresponding to polyforms $\beta_{1}^{\prime }$ and $\beta_{2}^{\prime }$ can be formed during spontaneous fermentation. The growth of CB crystals occurs in the form of rod/needle/fiber and plate, just like non-fermented beans. The results allow the fermentation process to be divided into two stages: the first fermentation stage until day 3 and the second stage from day 4 to day 7. Low crystallization rates are obtained in this last stage, so the cocoa butter crystallizes more slowly. This happens due to the formation of other chemical components within the cocoa bean due to the fermentation process.

## Figures and Tables

**Table 1:** Values for the Avrami index, *n*, for different types of nucleation and growth.

n	Type of Crystal Growth and Nucleation Expected
3 + 1 = 4	Spherical crystals grow from sporadic nuclei
3 + 0 = 3	Spherical crystals grow from instantaneous nuclei
2 + 1 = 3	Crystals grow plate-like from sporadic nuclei
2 + 0 = 2	Crystals grow plate-like from instantaneous nuclei
1 + 1 = 2	Crystals grow as rod/needle/fiber from sporadic nuclei
1 + 0 = 1	Crystals grow as rod/needle/fiber from instantaneous nuclei.

**Table 2:** Crystallization and melting profiles of CB and POP standard polymorphism at 5 °C min^−1^.

Sample	Crystallization				Melting				Polymorphic Form
	$T_{onset}$ (°C)	$T_{c}$ (°C)	$T_{endset}$ (°C)	Enthalpy (J/g)	$T_{onset}$ (°C)	$T_{m}$ (°C)	$T_{endset}$ (°C)	Enthalpy (J/g)	
CB	15.45	12.93	2.99	48.42	15.05	19.65	26.24	69.01	γ
POP	12.07	10.40	4.55	54.91	12.75	15.75	19.34	76.49	γ

**Table 3:** Kinetics crystallization of CB and POP standard at different $T_{c}$.

Crystallization Temperature (°C)	Sample	n	k (min^−n^)	$t_{0}$ (min)	$t_{1/2}\;theo$ (min)	$t_{1/2}\;exp$ (min)	$R^{2}$
15	CB	2.53	2.87 × 10^−3^	13.19	8.77	8.75	0.9990
	POP	3.07	2.33 × 10^−5^	5.89	28.54	28.57	0.9996
16	CB	2.91	5.97 × 10^−4^	14.59	11.30	11.42	0.9993
	POP	2.94	1.94 × 10^−5^	11.56	35.36	35.05	0.9920
17	CB	2.99	3.01 × 10^−4^	16.20	13.28	13.29	0.9992
	POP	2.84	2.56 × 10^−5^	14.33	36.18	36.70	0.9999
18	CB	3.42	9.70 × 10^−5^	15.61	13.36	13.74	0.9997
	POP	2.35	6.04 × 10^−5^	19.27	53.29	52.98	0.9998
19	CB	3.73	8.54 × 10^−5^	17.85	15.90	16.35	0.9993
	POP	2.61	1.06 × 10^−5^	35.27	70.13	72.17	0.9989

**Table 4:** Kinetics of crystallization parameters of CB inside cocoa beans during spontaneous fermentation.

Crystallization Temperature (°C)	Sample	n	k (min^−n^)	$t_{1/2}\;theo$ (min)	$t_{1/2}\;exp$ (min)	$R^{2}$
15	Non-fermented	2.13	1.11 × 10^−2^	6.99	6.55	0.9992
	Day 0	2.09	8.68 × 10^−3^	8.09	8.31	0.9984
	Day 1	2.66	1.04 ×10^−3^	11.55	12.33	0.9993
	Day 2	2.58	1.53 × 10^−3^	10.73	11.26	0.9994
	Day 3	1.76	2.44 × 10^−2^	6.69	7.10	0.9918
	Day 4	2.51	1.75 × 10^−3^	10.81	10.52	0.9995
	Day 5	2.76	8.06 × 10^−4^	11.57	11.90	0.9998
	Day 6	2.04	3.02 × 10^−3^	14.34	12.70	0.9999
	Day 7	2.34	3.32 × 10^−3^	9.83	9.75	0.9994
16	Non-fermented	1.51	2.56 × 10^−2^	8.93	7.77	0.9921
	Day 0	1.78	1.59 × 10^−2^	8.31	8.05	0.9973
	Day 1	2.13	2.38 × 10^−3^	14.54	14.98	0.9991
	Day 2	2.49	1.65 × 10^−3^	11.25	11.36	0.9998
	Day 3	2.00	1.42 × 10^−2^	6.96	6.93	0.9992
	Day 4	2.67	1.10 × 10^−3^	11.14	10.80	0.9977
	Day 5	2.27	1.09 × 10^−3^	17.24	16.43	0.9929
	Day 6	1.67	7.13 × 10^−3^	15.43	14.27	0.9903
	Day 7	2.80	6.37 × 10^−4^	11.14	12.60	0.9992
17	Non-fermented	1.92	6.82 × 10^−3^	11.14	10.58	0.9965
	Day 0	2.08	7.22 × 10^−3^	8.97	9.17	0.9991
	Day 1	1.82	3.10 × 10^−3^	15.64	12.35	0.9924
	Day 2	2.54	9.54 × 10^−4^	13.41	13.30	0.9986
	Day 3	2.43	2.93 × 10^−3^	9.51	9.93	0.9998
	Day 4	2.94	5.68 × 10^−4^	11.20	11.43	0.9999
	Day 5	2.47	1.77 × 10^−3^	11.24	11.90	0.9985
	Day 6	2.45	9.05 × 10^−4^	15.07	15.19	0.9990
	Day 7	2.35	1.49 × 10^−3^	13.61	13.87	0.9999
18	Non-fermented	2.10	3.61 × 10^−3^	12.23	11.73	0.9952
	Day 0	1.79	6.78 × 10^−3^	13.21	13.48	0.9936
	Day 1	1.73	8.77 × 10^−3^	12.56	13.28	0.9984
	Day 2	2.94	2.51 × 10^−4^	14.84	15.78	0.9994
	Day 3	2.55	1.98 × 10^−3^	9.91	10.14	0.9999
	Day 4	2.82	6.48 × 10^−4^	11.89	12.33	0.9998
	Day 5	2.55	8.59 × 10^−4^	13.74	14.24	0.9993
	Day 6	2.59	5.78 × 10^−4^	15.40	15.43	0.9993
	Day 7	1.98	3.45 × 10^−3^	14.63	13.94	0.9964
19	Non-fermented	2.17	3.01 × 10^−3^	12.29	12.28	0.9997
	Day 0	1.60	8.14 × 10^−3^	16.01	14.08	0.9862
	Day 1	1.20	9.38 × 10^−3^	12.02	12.24	0.9991
	Day 2	2.66	4.20 × 10^−4^	16.12	17.18	0.9989
	Day 3	2.09	2.75 × 10^−3^	14.09	15.68	0.9985
	Day 4	2.94	1.72 × 10^−4^	16.90	17.88	0.9981
	Day 5	2.29	1.66 × 10^−3^	13.89	14.55	0.9995
	Day 6	2.57	4.73 × 10^−4^	17.12	17.85	0.9996
	Day 7	2.68	3.88 × 10^−4^	16.30	16.96	0.9986

**Table 5:** Polymorphisms of CB and POP standard at different $T_{c}$.

Crystallization Temperature (°C)	Sample	Enthalpy (J/g)	$T_{onset}$ (°C)	$T_{m}$ (°C)	$T_{endset}$ (°C)	Polymorphic Form
15	CB	89.53	19.82	25.80	28.59	$\beta_{1}^{\prime }$
	POP	105.92	20.88	24.70	27.34	α
16	CB	87.16	20.48	26.27	28.80	$\beta_{1}^{\prime }$
	POP	105.06	21.38	25.92	27.71	$\beta_{2}^{\prime }$
17	CB	84.06	20.79	26.69	28.93	$\beta_{1}^{\prime }$
	POP	104.67	23.18	26.66	28.29	$\beta_{2}^{\prime }$
18	CB	75.80	21.42	27.01	29.04	$\beta_{1}^{\prime }$
	POP	103.20	23.46	26.17	27.87	$\beta_{2}^{\prime }$
19	CB	69.73	22.14	27.42	29.30	$\beta_{1}^{\prime }$
	POP	80.23	24.25	26.56	28.09	$\beta_{2}^{\prime }$

**Table 6:** Polymorphism of CB inside cocoa beans during spontaneous fermentation.

Crystallization Temperature (°C)	Sample	Enthalpy (J/g)	$T_{onset}$ (°C)	$T_{m}$ (°C)	$T_{endset}$ (°C)	Polymorphic Form
15	Non-fermented	23.42	20.31	26.19	29.29	$\beta_{1}^{\prime }$
	Day 0	33.81	20.66	26.32	30.22	$\beta_{1}^{\prime }$
	Day 1	17.53	19.14	24.72	28.17	$\beta_{2}^{\prime }$
	Day 2	34.89	20.26	25.73	28.76	$\beta_{1}^{\prime }$
	Day 3	33.33	19.55	24.84	28.17	$\beta_{2}^{\prime }$
	Day 4	32.55	19.62	25.42	29.04	$\beta_{1}^{\prime }$
	Day 5	32.94	20.65	26.14	29.59	$\beta_{1}^{\prime }$
	Day 6	36.58	20.81	26.96	30.01	$\beta_{1}^{\prime }$
	Day 7	22.88	19.06	24.44	27.08	$\beta_{2}^{\prime }$
16	Non-fermented	18.75	21.07	26.41	29.54	$\beta_{1}^{\prime }$
	Day 0	17.69	22.38	26.48	28.81	$\beta_{1}^{\prime }$
	Day 1	14.92	20.02	24.90	28.22	$\beta_{2}^{\prime }$
	Day 2	33.92	20.78	25.79	28.67	$\beta_{1}^{\prime }$
	Day 3	27.16	19.90	25.42	28.18	$\beta_{2}^{\prime }$
	Day 4	30.88	20.14	25.69	29.07	$\beta_{1}^{\prime }$
	Day 5	32.27	20.77	26.42	29.63	$\beta_{1}^{\prime }$
	Day 6	31.99	21.90	27.10	30.35	$\beta_{1}^{\prime }$
	Day 7	21.21	19.81	24.64	27.18	$\beta_{2}^{\prime }$
17	Non-fermented	16.19	21.40	26.69	29.86	$\beta_{1}^{\prime }$
	Day 0	12.38	22.05	26.56	28.94	$\beta_{1}^{\prime }$
	Day 1	11.61	20.83	25.28	28.28	$\beta_{2}^{\prime }$
	Day 2	33.19	21.25	25.97	28.70	$\beta_{1}^{\prime }$
	Day 3	22.61	20.81	25.82	28.34	$\beta_{1}^{\prime }$
	Day 4	28.87	20.89	26.03	29.34	$\beta_{1}^{\prime }$
	Day 5	31.42	21.22	26.54	29.63	$\beta_{1}^{\prime }$
	Day 6	30.35	21.17	25.78	28.49	$\beta_{1}^{\prime }$
	Day 7	18.95	20.53	24.97	27.16	$\beta_{2}^{\prime }$
18	Non-fermented	14.17	22.20	27.15	30.06	$\beta_{1}^{\prime }$
	Day 0	9.16	22.57	26.79	29.31	$\beta_{1}^{\prime }$
	Day 1	7.53	21.66	25.77	28.37	$\beta_{1}^{\prime }$
	Day 2	28.42	22.11	26.26	28.90	$\beta_{1}^{\prime }$
	Day 3	18.28	21.83	26.10	28.44	$\beta_{1}^{\prime }$
	Day 4	26.78	21.73	26.38	29.63	$\beta_{1}^{\prime }$
	Day 5	30.53	21.78	26.79	29.64	$\beta_{1}^{\prime }$
	Day 6	29.60	21.85	26.20	28.62	$\beta_{1}^{\prime }$
	Day 7	16.14	21.27	25.30	27.29	$\beta_{2}^{\prime }$
19	Non-fermented	11.21	23.19	27.63	30.30	$\beta_{1}^{\prime }$
	Day 0	6.25	23.26	27.06	29.52	$\beta_{1}^{\prime }$
	Day 1	4.01	22.42	26.42	28.65	$\beta_{1}^{\prime }$
	Day 2	23.94	22.46	26.55	28.97	$\beta_{1}^{\prime }$
	Day 3	13.86	22.48	26.48	28.69	$\beta_{1}^{\prime }$
	Day 4	23.45	22.12	26.83	29.66	$\beta_{1}^{\prime }$
	Day 5	26.83	22.48	27.22	29.78	$\beta_{1}^{\prime }$
	Day 6	27.27	22.35	26.62	28.82	$\beta_{1}^{\prime }$
	Day 7	12.12	21.82	25.98	27.61	$\beta_{1}^{\prime }$

## Data Availability

The data presented in this study are available in Supplementary Material.

## Supplementary Materials

The following are available online at https://www.mdpi.com/article/10.3390/foods11121769/s1, Tables S1 and S2 shows the POP and CB isothermal crystallization data, respectively.
